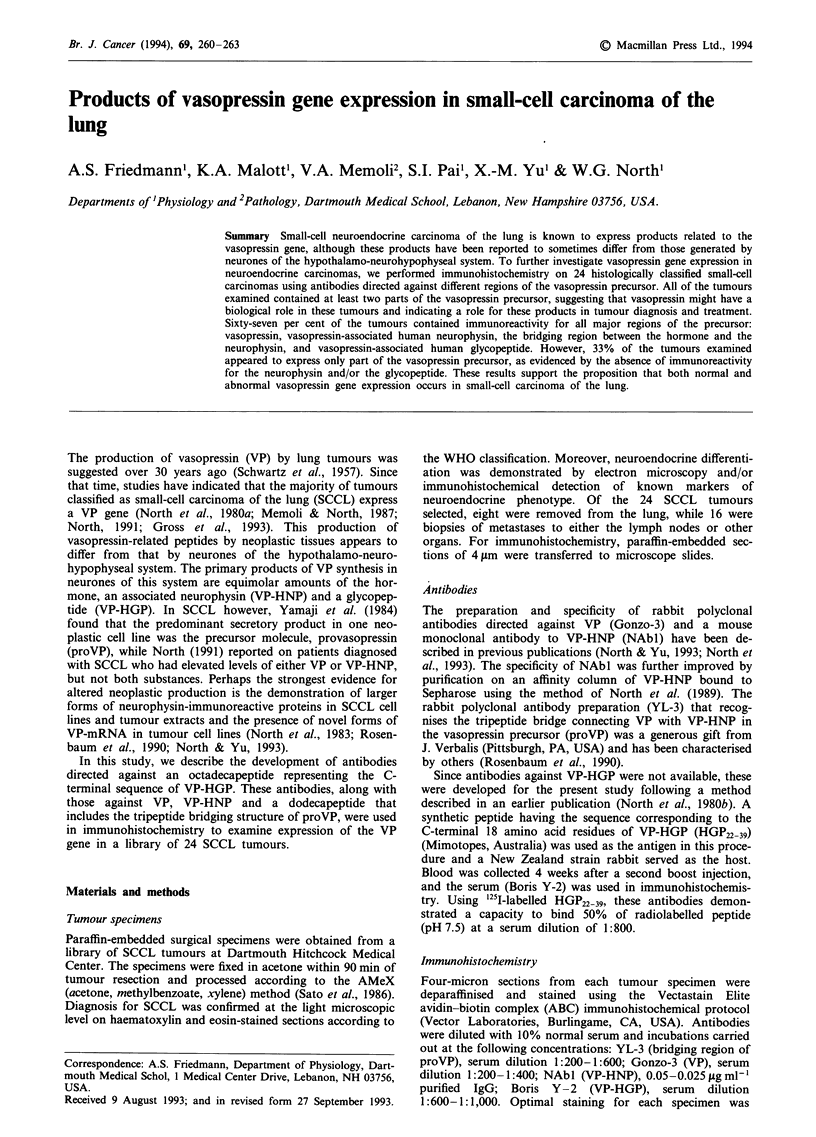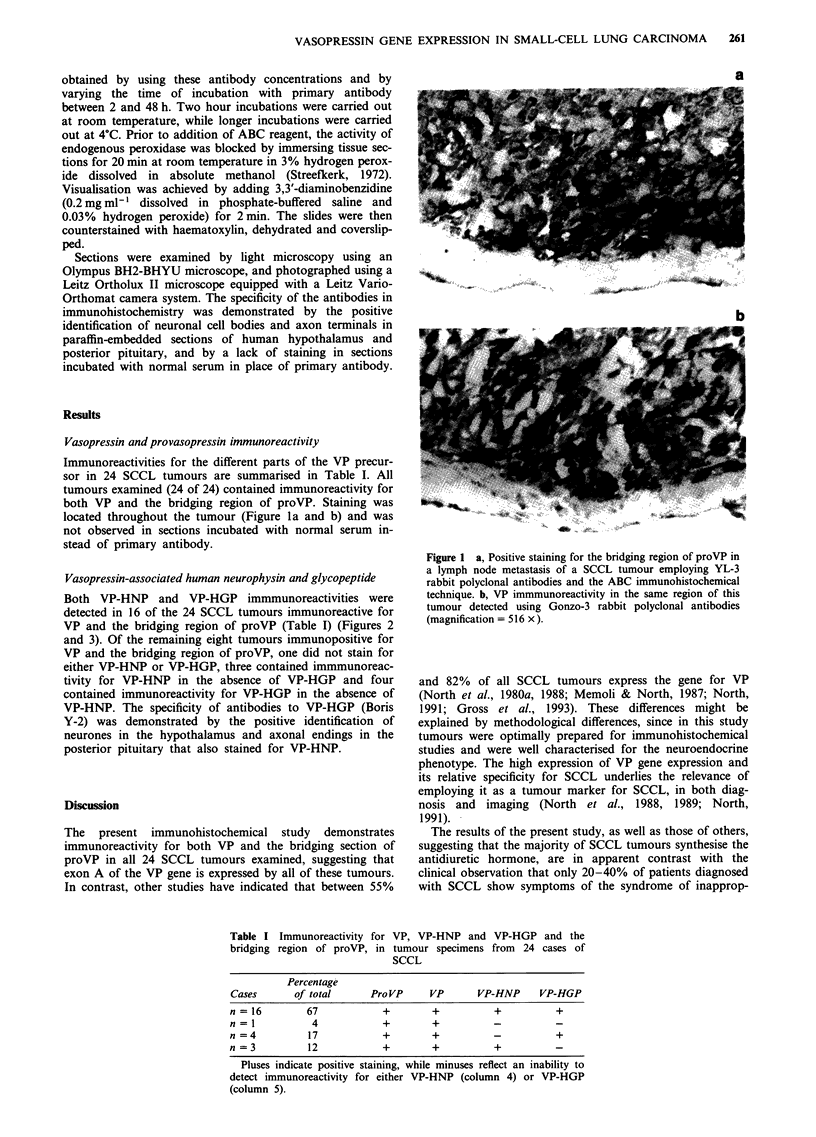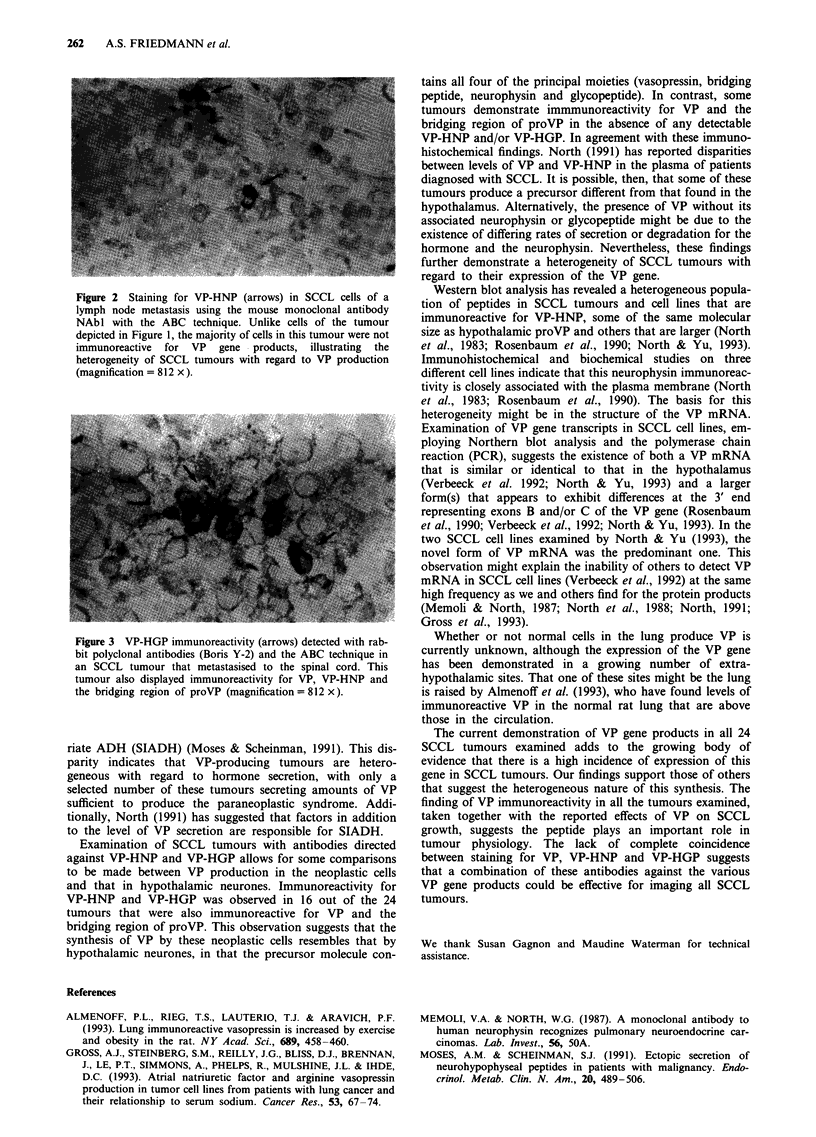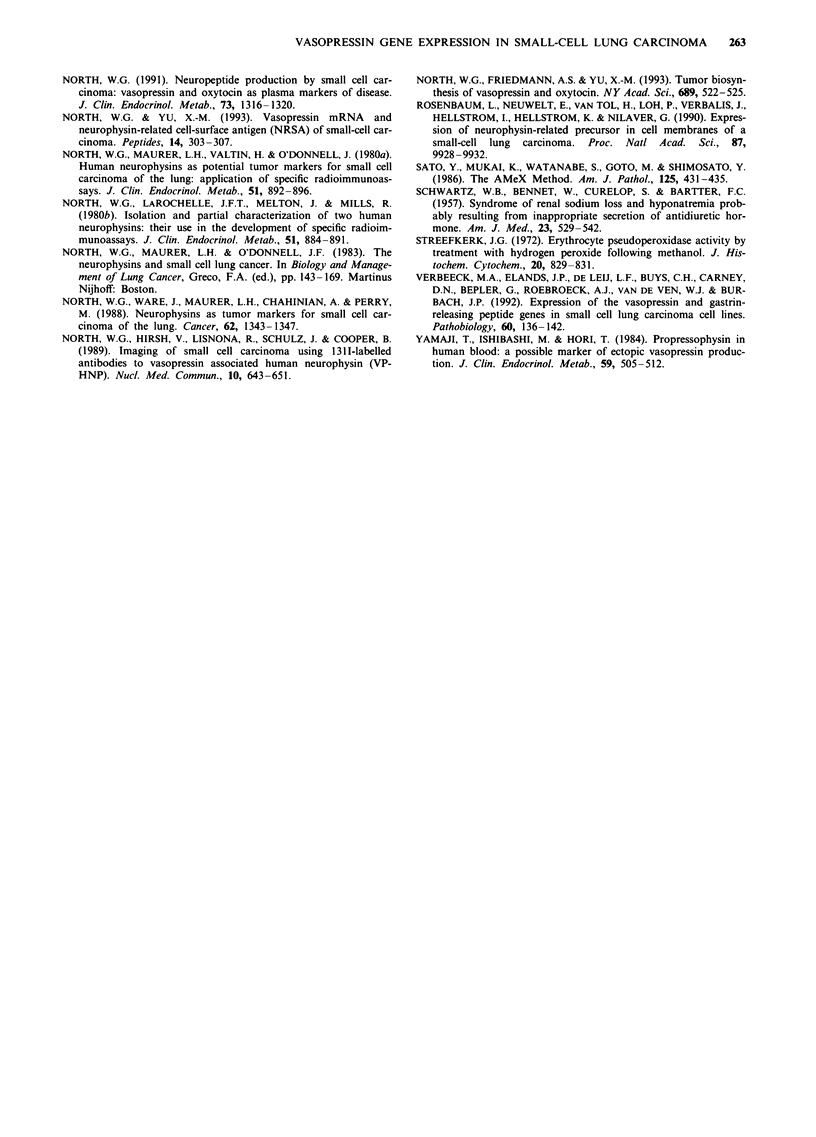# Products of vasopressin gene expression in small-cell carcinoma of the lung.

**DOI:** 10.1038/bjc.1994.49

**Published:** 1994-02

**Authors:** A. S. Friedmann, K. A. Malott, V. A. Memoli, S. I. Pai, X. M. Yu, W. G. North

**Affiliations:** Department of Physiology, Dartmouth Medical School, Lebanon, New Hampshire 03756.

## Abstract

**Images:**


Br. J. Cancer (1994), 69, 260-263 © Macmillan Press Ltd., 1994

### Products of vasopressin gene expression in small-cell carcinoma of the lung

A.S. Friedmann[1], K.A. Malott[1], V.A. Memoli[2], S.I. Pai[1], X.-M. Yu[1] & W.G. North[1]

*Departments of [1]Physiology and [2]Pathology, Dartmouth Medical School, Lebanon, New Hampshire 03756, USA.*

**Summary** Small-cell neuroendocrine carcinoma of the lung is known to express products related to the vasopressin gene, although these products have been reported to sometimes differ from those generated by neurones of the hypothalamo-neurohypophyseal system. To further investigate vasopressin gene expression in neuroendocrine carcinomas, we performed immunohistochemistry on 24 histologically classified small-cell carcinomas using antibodies directed against different regions of the vasopressin precursor. All of the tumours examined contained at least two parts of the vasopressin precursor, suggesting that vasopressin might have a biological role in these tumours and indicating a role for these products in tumour diagnosis and treatment. Sixty-seven per cent of the tumours contained immunoreactivity for all major regions of the precursor: vasopressin, vasopressin-associated human neurophysin, the bridging region between the hormone and the neurophysin, and vasopressin-associated human glycopeptide. However, 33% of the tumours examined appeared to express only part of the vasopressin precursor, as evidenced by the absence of immunoreactivity for the neurophysin and/or the glycopeptide. These results support the proposition that both normal and abnormal vasopressin gene expression occurs in small-cell carcinoma of the lung.

The production of vasopressin (VP) by lung tumours was suggested over 30 years ago (Schwartz *et al.*, 1957). Since that time, studies have indicated that the majority of tumours classified as small-cell carcinoma of the lung (SCCL) express a VP gene (North *et al.*, 1980*a*; Memoli & North, 1987; North, 1991; Gross *et al.*, 1993). This production of vasopressin-related peptides by neoplastic tissues appears to differ from that by neurones of the hypothalamo-neurohypophyseal system. The primary products of VP synthesis in neurones of this system are equimolar amounts of the hormone, an associated neurophysin (VP-HNP) and a glycopeptide (VP-HGP). In SCCL however, Yamaji *et al.* (1984) found that the predominant secretory product in one neoplastic cell line was the precursor molecule, provasopressin (proVP), while North (1991) reported on patients diagnosed with SCCL who had elevated levels of either VP or VP-HNP, but not both substances. Perhaps the strongest evidence for altered neoplastic production is the demonstration of larger forms of neurophysin-immunoreactive proteins in SCCL cell lines and tumour extracts and the presence of novel forms of VP-mRNA in tumour cell lines (North *et al.*, 1983; Rosenbaum *et al.*, 1990; North & Yu, 1993).

In this study, we describe the development of antibodies directed against an octadecapeptide representing the C-terminal sequence of VP-HGP. These antibodies, along with those against VP, VP-HNP and a dodecapeptide that includes the tripeptide bridging structure of proVP, were used in immunohistochemistry to examine expression of the VP gene in a library of 24 SCCL tumours.

### Materials and methods

### *Tumour specimens*

Paraffin-embedded surgical specimens were obtained from a library of SCCL tumours at Dartmouth Hitchcock Medical Center. The specimens were fixed in acetone within 90 min of tumour resection and processed according to the AMeX (*a*cetone, *m*ethylbenzoate, *x*ylene) method (Sato *et al.*, 1986). Diagnosis for SCCL was confirmed at the light microscopic level on haematoxylin and eosin-stained sections according to the WHO classification. Moreover, neuroendocrine differentiation was demonstrated by electron microscopy and/or immunohistochemical detection of known markers of neuroendocrine phenotype. Of the 24 SCCL tumours selected, eight were removed from the lung, while 16 were biopsies of metastases to either the lymph nodes or other organs. For immunohistochemistry, paraffin-embedded sections of 4 μm were transferred to microscope slides.

### *Antibodies*

The preparation and specificity of rabbit polyclonal antibodies directed against VP (Gonzo-3) and a mouse monoclonal antibody to VP-HNP (NAb1) have been described in previous publications (North & Yu, 1993; North *et al.*, 1993). The specificity of NAb1 was further improved by purification on an affinity column of VP-HNP bound to Sepharose using the method of North *et al.* (1989). The rabbit polyclonal antibody preparation (YL-3) that recognises the tripeptide bridge connecting VP with VP-HNP in the vasopressin precursor (proVP) was a generous gift from J. Verbalis (Pittsburgh, PA, USA) and has been characterised by others (Rosenbaum *et al.*, 1990).

Since antibodies against VP-HGP were not available, these were developed for the present study following a method described in an earlier publication (North *et al.*, 1980*b*). A synthetic peptide having the sequence corresponding to the C-terminal 18 amino acid residues of VP-HGP ($HGP_{22-39}$) (Mimotopes, Australia) was used as the antigen in this procedure and a New Zealand strain rabbit served as the host. Blood was collected 4 weeks after a second boost injection, and the serum (Boris Y-2) was used in immunohistochemistry. Using $^{125}$I-labelled $HGP_{22-39}$, these antibodies demonstrated a capacity to bind 50% of radiolabelled peptide (pH 7.5) at a serum dilution of 1:800.

### *Immunohistochemistry*

Four-micron sections from each tumour specimen were deparaffinised and stained using the Vectastain Elite avidin–biotin complex (ABC) immunohistochemical protocol (Vector Laboratories, Burlingame, CA, USA). Antibodies were diluted with 10% normal serum and incubations carried out at the following concentrations: YL-3 (bridging region of proVP), serum dilution 1:200–1:600; Gonzo-3 (VP), serum dilution 1:200–1:400; NAb1 (VP-HNP), 0.05–0.025 μg $ml^{-1}$ purified IgG; Boris Y-2 (VP-HGP), serum dilution 1:600–1:1,000. Optimal staining for each specimen was

Correspondence: A.S. Friedmann, Department of Physiology, Dartmouth Medical Schol, 1 Medical Center Drive, Lebanon, NH 03756, USA.
Received 9 August 1993; and in revised form 27 September 1993.

obtained by using these antibody concentrations and by varying the time of incubation with primary antibody between 2 and 48 h. Two hour incubations were carried out at room temperature, while longer incubations were carried out at 4°C. Prior to addition of ABC reagent, the activity of endogenous peroxidase was blocked by immersing tissue sections for 20 min at room temperature in 3% hydrogen peroxide dissolved in absolute methanol (Streefkerk, 1972). Visualisation was achieved by adding 3,3′-diaminobenzidine (0.2 mg $ml^{-1}$ dissolved in phosphate-buffered saline and 0.03% hydrogen peroxide) for 2 min. The slides were then counterstained with haematoxylin, dehydrated and coverslipped.

Sections were examined by light microscopy using an Olympus BH2-BHYU microscope, and photographed using a Leitz Ortholux II microscope equipped with a Leitz Vario-Orthomat camera system. The specificity of the antibodies in immunohistochemistry was demonstrated by the positive identification of neuronal cell bodies and axon terminals in paraffin-embedded sections of human hypothalamus and posterior pituitary, and by a lack of staining in sections incubated with normal serum in place of primary antibody.

### Results

*Vasopressin and provasopressin immunoreactivity*

Immunoreactivities for the different parts of the VP precursor in 24 SCCL tumours are summarised in Table I. All tumours examined (24 of 24) contained immunoreactivity for both VP and the bridging region of proVP. Staining was located throughout the tumour (Figure 1a and b) and was not observed in sections incubated with normal serum instead of primary antibody.

*Vasopressin-associated human neurophysin and glycopeptide*

Both VP-HNP and VP-HGP immmunoreactivities were detected in 16 of the 24 SCCL tumours immunoreactive for VP and the bridging region of proVP (Table I) (Figures 2 and 3). Of the remaining eight tumours immunopositive for VP and the bridging region of proVP, one did not stain for either VP-HNP or VP-HGP, three contained immmunoreactivity for VP-HNP in the absence of VP-HGP and four contained immunoreactivity for VP-HGP in the absence of VP-HNP. The specificity of antibodies to VP-HGP (Boris Y-2) was demonstrated by the positive identification of neurones in the hypothalamus and axonal endings in the posterior pituitary that also stained for VP-HNP.

### Discussion

The present immunohistochemical study demonstrates immunoreactivity for both VP and the bridging section of proVP in all 24 SCCL tumours examined, suggesting that exon A of the VP gene is expressed by all of these tumours. In contrast, other studies have indicated that between 55% and 82% of all SCCL tumours express the gene for VP (North *et al.*, 1980*a*, 1988; Memoli & North, 1987; North, 1991; Gross *et al.*, 1993). These differences might be explained by methodological differences, since in this study tumours were optimally prepared for immunohistochemical studies and were well characterised for the neuroendocrine phenotype. The high expression of VP gene expression and its relative specificity for SCCL underlies the relevance of employing it as a tumour marker for SCCL, in both diagnosis and imaging (North *et al.*, 1988, 1989; North, 1991).

The results of the present study, as well as those of others, suggesting that the majority of SCCL tumours synthesise the antidiuretic hormone, are in apparent contrast with the clinical observation that only 20–40% of patients diagnosed with SCCL show symptoms of the syndrome of inapprop-

**Figure 1** **a**, Positive staining for the bridging region of proVP in a lymph node metastasis of a SCCL tumour employing YL-3 rabbit polyclonal antibodies and the ABC immunohistochemical technique. **b**, VP immmunoreactivity in the same region of this tumour detected using Gonzo-3 rabbit polyclonal antibodies (magnification = 516 ×).

**Table I** Immunoreactivity for VP, VP-HNP and VP-HGP and the bridging region of proVP, in tumour specimens from 24 cases of SCCL

| *Cases* | *Percentage of total* | *ProVP* | *VP* | *VP-HNP* | *VP-HGP* |
|---|---|---|---|---|---|
| $n = 16$ | 67 | + | + | + | + |
| $n = 1$ | 4 | + | + | − | − |
| $n = 4$ | 17 | + | + | − | + |
| $n = 3$ | 12 | + | + | + | − |

Pluses indicate positive staining, while minuses reflect an inability to detect immunoreactivity for either VP-HNP (column 4) or VP-HGP (column 5).

**Figure 2** Staining for VP-HNP (arrows) in SCCL cells of a lymph node metastasis using the mouse monoclonal antibody NAb1 with the ABC technique. Unlike cells of the tumour depicted in Figure 1, the majority of cells in this tumour were not immunoreactive for VP gene products, illustrating the heterogeneity of SCCL tumours with regard to VP production (magnification = 812 ×).

**Figure 3** VP-HGP immunoreactivity (arrows) detected with rabbit polyclonal antibodies (Boris Y-2) and the ABC technique in an SCCL tumour that metastasised to the spinal cord. This tumour also displayed immunoreactivity for VP, VP-HNP and the bridging region of proVP (magnification = 812 ×).

riate ADH (SIADH) (Moses & Scheinman, 1991). This disparity indicates that VP-producing tumours are heterogeneous with regard to hormone secretion, with only a selected number of these tumours secreting amounts of VP sufficient to produce the paraneoplastic syndrome. Additionally, North (1991) has suggested that factors in addition to the level of VP secretion are responsible for SIADH.

Examination of SCCL tumours with antibodies directed against VP-HNP and VP-HGP allows for some comparisons to be made between VP production in the neoplastic cells and that in hypothalamic neurones. Immunoreactivity for VP-HNP and VP-HGP was observed in 16 out of the 24 tumours that were also immunoreactive for VP and the bridging region of proVP. This observation suggests that the synthesis of VP by these neoplastic cells resembles that by hypothalamic neurones, in that the precursor molecule contains all four of the principal moieties (vasopressin, bridging peptide, neurophysin and glycopeptide). In contrast, some tumours demonstrate immmunoreactivity for VP and the bridging region of proVP in the absence of any detectable VP-HNP and/or VP-HGP. In agreement with these immunohistochemical findings. North (1991) has reported disparities between levels of VP and VP-HNP in the plasma of patients diagnosed with SCCL. It is possible, then, that some of these tumours produce a precursor different from that found in the hypothalamus. Alternatively, the presence of VP without its associated neurophysin or glycopeptide might be due to the existence of differing rates of secretion or degradation for the hormone and the neurophysin. Nevertheless, these findings further demonstrate a heterogeneity of SCCL tumours with regard to their expression of the VP gene.

Western blot analysis has revealed a heterogeneous population of peptides in SCCL tumours and cell lines that are immunoreactive for VP-HNP, some of the same molecular size as hypothalamic proVP and others that are larger (North et al., 1983; Rosenbaum et al., 1990; North & Yu, 1993). Immunohistochemical and biochemical studies on three different cell lines indicate that this neurophysin immunoreactivity is closely associated with the plasma membrane (North et al., 1983; Rosenbaum et al., 1990). The basis for this heterogeneity might be in the structure of the VP mRNA. Examination of VP gene transcripts in SCCL cell lines, employing Northern blot analysis and the polymerase chain reaction (PCR), suggests the existence of both a VP mRNA that is similar or identical to that in the hypothalamus (Verbeeck et al. 1992; North & Yu, 1993) and a larger form(s) that appears to exhibit differences at the 3′ end representing exons B and/or C of the VP gene (Rosenbaum et al., 1990; Verbeeck et al., 1992; North & Yu, 1993). In the two SCCL cell lines examined by North & Yu (1993), the novel form of VP mRNA was the predominant one. This observation might explain the inability of others to detect VP mRNA in SCCL cell lines (Verbeeck et al., 1992) at the same high frequency as we and others find for the protein products (Memoli & North, 1987; North et al., 1988; North, 1991; Gross et al., 1993).

Whether or not normal cells in the lung produce VP is currently unknown, although the expression of the VP gene has been demonstrated in a growing number of extra-hypothalamic sites. That one of these sites might be the lung is raised by Almenoff et al. (1993), who have found levels of immunoreactive VP in the normal rat lung that are above those in the circulation.

The current demonstration of VP gene products in all 24 SCCL tumours examined adds to the growing body of evidence that there is a high incidence of expression of this gene in SCCL tumours. Our findings support those of others that suggest the heterogeneous nature of this synthesis. The finding of VP immunoreactivity in all the tumours examined, taken together with the reported effects of VP on SCCL growth, suggests the peptide plays an important role in tumour physiology. The lack of complete coincidence between staining for VP, VP-HNP and VP-HGP suggests that a combination of these antibodies against the various VP gene products could be effective for imaging all SCCL tumours.

We thank Susan Gagnon and Maudine Waterman for technical assistance.

### References

ALMENOFF, P.L., RIEG, T.S., LAUTERIO, T.J. & ARAVICH, P.F. (1993). Lung immunoreactive vasopressin is increased by exercise and obesity in the rat. *NY Acad. Sci.*, **689**, 458–460.

GROSS, A.J., STEINBERG, S.M., REILLY, J.G., BLISS, D.J., BRENNAN, J., LE, P.T., SIMMONS, A., PHELPS, R., MULSHINE, J.L. & IHDE, D.C. (1993). Atrial natriuretic factor and arginine vasopressin production in tumor cell lines from patients with lung cancer and their relationship to serum sodium. *Cancer Res.*, **53**, 67–74.

MEMOLI, V.A. & NORTH, W.G. (1987). A monoclonal antibody to human neurophysin recognizes pulmonary neuroendocrine carcinomas. *Lab. Invest.*, **56**, 50A.

MOSES, A.M. & SCHEINMAN, S.J. (1991). Ectopic secretion of neurohypophyseal peptides in patients with malignancy. *Endocrinol. Metab. Clin. N. Am.*, **20**, 489–506.

NORTH, W.G. (1991). Neuropeptide production by small cell carcinoma: vasopressin and oxytocin as plasma markers of disease. *J. Clin. Endocrinol. Metab.*, **73**, 1316–1320.

NORTH, W.G. & YU, X.-M. (1993). Vasopressin mRNA and neurophysin-related cell-surface antigen (NRSA) of small-cell carcinoma. *Peptides*, **14**, 303–307.

NORTH, W.G., MAURER, L.H., VALTIN, H. & O'DONNELL, J. (1980*a*). Human neurophysins as potential tumor markers for small cell carcinoma of the lung: application of specific radioimmunoassays. *J. Clin. Endocrinol. Metab.*, **51**, 892–896.

NORTH, W.G., LAROCHELLE, J.F.T., MELTON, J. & MILLS, R. (1980*b*). Isolation and partial characterization of two human neurophysins: their use in the development of specific radioimmunoassays. *J. Clin. Endocrinol. Metab.*, **51**, 884–891.

NORTH, W.G., MAURER, L.H. & O'DONNELL, J.F. (1983). The neurophysins and small cell lung cancer. In *Biology and Management of Lung Cancer*, Greco, F.A. (ed.), pp. 143–169. Martinus Nijhoff: Boston.

NORTH, W.G., WARE, J., MAURER, L.H., CHAHINIAN, A. & PERRY, M. (1988). Neurophysins as tumor markers for small cell carcinoma of the lung. *Cancer*, **62**, 1343–1347.

NORTH, W.G., HIRSH, V., LISNONA, R., SCHULZ, J. & COOPER, B. (1989). Imaging of small cell carcinoma using 131I-labelled antibodies to vasopressin associated human neurophysin (VP-HNP). *Nucl. Med. Commun.*, **10**, 643–651.

NORTH, W.G., FRIEDMANN, A.S. & YU, X.-M. (1993). Tumor biosynthesis of vasopressin and oxytocin. *NY Acad. Sci.*, **689**, 522–525.

ROSENBAUM, L., NEUWELT, E., VAN TOL, H., LOH, P., VERBALIS, J., HELLSTROM, I., HELLSTROM, K. & NILAVER, G. (1990). Expression of neurophysin-related precursor in cell membranes of a small-cell lung carcinoma. *Proc. Natl Acad. Sci.*, **87**, 9928–9932.

SATO, Y., MUKAI, K., WATANABE, S., GOTO, M. & SHIMOSATO, Y. (1986). The AMeX Method. *Am. J. Pathol.*, **125**, 431–435.

SCHWARTZ, W.B., BENNET, W., CURELOP, S. & BARTTER, F.C. (1957). Syndrome of renal sodium loss and hyponatremia probably resulting from inappropriate secretion of antidiuretic hormone. *Am. J. Med.*, **23**, 529–542.

STREEFKERK, J.G. (1972). Erythrocyte pseudoperoxidase activity by treatment with hydrogen peroxide following methanol. *J. Histochem. Cytochem.*, **20**, 829–831.

VERBEECK, M.A., ELANDS, J.P., DE LEIJ, L.F., BUYS, C.H., CARNEY, D.N., BEPLER, G., ROEBROECK, A.J., VAN DE VEN, W.J. & BURBACH, J.P. (1992). Expression of the vasopressin and gastrin-releasing peptide genes in small cell lung carcinoma cell lines. *Pathobiology*, **60**, 136–142.

YAMAJI, T., ISHIBASHI, M. & HORI, T. (1984). Propressophysin in human blood: a possible marker of ectopic vasopressin production. *J. Clin. Endocrinol. Metab.*, **59**, 505–512.